# Design, implementation, and evaluation of the computer-aided clinical decision support system based on learning-to-rank: collaboration between physicians and machine learning in the differential diagnosis process

**DOI:** 10.1186/s12911-023-02123-5

**Published:** 2023-02-02

**Authors:** Yasuhiko Miyachi, Osamu Ishii, Keijiro Torigoe

**Affiliations:** 1 The Society for Computer-Aided Clinical Decision Support System, Torigoe Clinic, Ibara, Okayama Japan; 2 Torigoe Clinic, Ibara, Okayama Japan

**Keywords:** Clinical decision support system, Diagnosis decision support system, Differential diagnosis, Diagnostic error, Rare diseases, Information retrieval, Learning to rank, Listwise approach, Approximate NDCG, Explainable artificial intelligence

## Abstract

**Background:**

We are researching, developing, and publishing the clinical decision support system based on learning-to-rank. The main objectives are (1) To support for differential diagnoses performed by internists and general practitioners and (2) To prevent diagnostic errors made by physicians. The main features are that “A physician inputs a patient's symptoms, findings, and test results to the system, and the system outputs a ranking list of possible diseases”.

**Method:**

The software libraries for machine learning and artificial intelligence are TensorFlow and TensorFlow Ranking. The prediction algorithm is Learning-to-Rank with the listwise approach. The ranking metric is normalized discounted cumulative gain (NDCG). The loss functions are Approximate NDCG (A-NDCG). We evaluated the machine learning performance on k-fold cross-validation. We evaluated the differential diagnosis performance with validated cases.

**Results:**

The machine learning performance of our system was much higher than that of the conventional system. The differential diagnosis performance of our system was much higher than that of the conventional system. We have shown that the clinical decision support system prevents physicians' diagnostic errors due to confirmation bias.

**Conclusions:**

We have demonstrated that the clinical decision support system is useful for supporting differential diagnoses and preventing diagnostic errors. We propose that differential diagnosis by physicians and learning-to-rank by machine has a high affinity. We found that information retrieval and clinical decision support systems have much in common (Target data, learning-to-rank, etc.). We propose that Clinical Decision Support Systems have the potential to support: (1) recall of rare diseases, (2) differential diagnoses for difficult-to-diagnoses cases, and (3) prevention of diagnostic errors. Our system can potentially evolve into an explainable clinical decision support system.

**Supplementary Information:**

The online version contains supplementary material available at 10.1186/s12911-023-02123-5.

## Introduction

We are researching, developing, and publishing the Clinical Decision Support System (CDSS) based on Learning-to-Rank (LTR) [1, 2].

This paper discusses our system's design, implementation, and evaluation.

### Diagnostic errors and clinical decision support system

Medical errors are among the most critical safety issues in today's healthcare. Medical errors cause the most significant damage (human and economic) to the public.

The well-known report "To Err Is Human." reports that 44,000–98,000 patients die annually in the United States due to medical errors. Deaths due to medical errors are more incredible than deaths due to the three leading causes of death (automobile accidents, breast cancer, and AIDS) [3].

Diagnostic errors are a type of medical error.

Briefly, diagnostic errors are as follows:

- A delayed diagnosis
- A wrong diagnosis
- A missed diagnosis [4]

The CDSS will be a competent partner with physicians to prevent diagnostic errors.

In clinical practice, internists and general practitioners also want the practical application of CDSS [5].

### Rare diseases, difficult-to-diagnose cases, and clinical diagnosis support systems

Rare diseases (RD) are a generic term for diseases with small patient populations. Rare diseases are the antonym of Common diseases. The definition of rare diseases and the criteria for prevalence are different for each country.

Table 1 shows the Definitions of rare diseases for each country.

**Table 1:** Definitions of rare diseases for each country

Country	Prevalence	Source
The EU	< 1 person in 2000	EU research on rare diseases
Japan	Not defined	Act on Medical Care for Patients with Intractable Diseases
The UK	< 1 person in 2000	The UK Rare Diseases Framework
The US	< 50,000 persons in the US	Rare Diseases Act of 2002

Difficult-to-diagnose cases have no formal definition. For example, many case reports describe difficult-to-diagnose cases. Rare diseases are often difficult-to-diagnose cases.

Various leading researchers have reported the application of the CDSS for the diagnosis of RD [6, 7].

### Main objectives of the clinical decision support system

In our study, the main objectives of the Clinical Decision Support System (CDSS) are as follows:

- To support differential diagnoses performed by internists and general practitioners.
- To prevent diagnostic errors made by physicians

### Main features of the clinical decision support system

In our study, the main features of the Clinical Decision Support System (CDSS) are as follows:

**A physician inputs a patient’s symptoms, findings, and test results to the system, and the system outputs a ranking list of possible diseases.**

The input information is as follows:

- Subjective symptoms
- Objective findings
- Physical findings
- Laboratory test results
- Imaging test results
- Other Information

(From now on, referred to as "inputted symptoms").

The output information is as follows:

- A ranking list of possible diseases

(From now on, referred to as "predicted diseases").

Clinical Decision Support system (CDSS) for Differential Diagnosis (DDx) is also known as Diagnostic Decision Support System (DDSS) [8].

### Example of the clinical decision support system

**Fig. 1:**
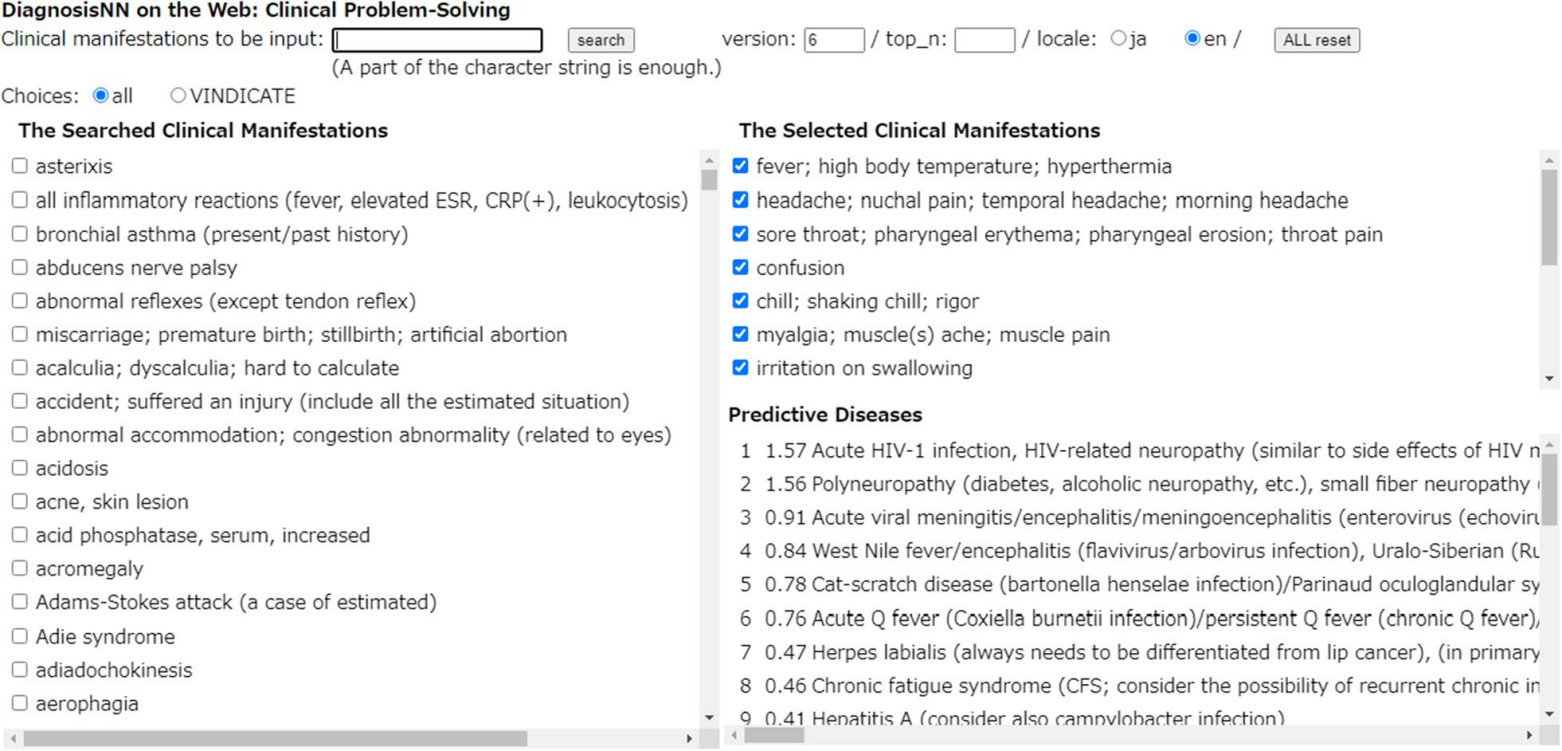
Example of the prediction screen of our system For details, see “Difficult-to-diagnose case with few characteristic symptoms” section. Difficult-to-driagnose case with few characteristic symptoms

Figure 1 shows the Example of the prediction screen of our system.

Table 2 shows the Example of the predicted results of our system.

**Table 2:** Example of the predicted results of our system

	Inputted symptoms			Score	Predicted diseases
a	Fever		1	1.61	Acute HIV-1 infection
b	Headache		2	1.51	Polyneuropathy
c	Sore throat		3	0.91	Acute viral meningitis
d	Consciousness indistinctness		4	0.88	West Nile fever
e	Chills		5	0.77	Cat-scratch disease
f	Muscles ache		6	0.46	Acute Q fever
g	Swallowing pain	→	7	0.23	Hepatitis A
h	Pharyngolaryngeal abnormality		8	0.21	Chronic fatigue syndrome
i	Aphasia		9	0.13	Sepsis
j	Apraxia		10	0.12	Toxoplasmosis
k	Fatigue				…
l	Muscle weakness				
m	Anorexia				
n	Weight loss				
o	Dementia				

For details, see: “Difficult-to-diagnose case with few characteristic symptoms” section

On the Internet, our system is open to healthcare professionals.

### Figures and tables

(See Tables 1, 2 and Fig. 1).

## Background

### Differential diagnosis process by physicians and learning-to-rank by machines

The Differential Diagnosis (DDx) process by experienced physicians is an iterative process with the following steps:

1. Perform medical examinations to obtain information about the diseases.
2. Recall multiple differential diseases.
3. Refine the recalled differential diseases.
4. Rank the refined differential diseases [9].

Learning-to-Rank (LTR) is a Machine Learning (ML) framework.

LTR is used to construct ranking models for Information Retrieval (IR) systems, recommendation systems, collaborative filtering systems, etc. [10].

We propose that the DDx process by experienced physicians is highly affinitive to LTR by machines.

LTR includes the following approaches:

- Pointwise approach
- Pairwise approach
- Listwise approach [10]

From the perspective of LTR, the DDx process by experienced physicians IS NOT a pointwise or pairwise approach.

- Pointwise approach:
  - Score one differential disease at a time.
- Pairwise approach:
  - Compare two differential diseases at a time.

This process IS a listwise approach.

- Listwise approach:
  1. Recall multiple differential diseases
  2. Refine the recalled differential diseases
  3. Rank the refined differential diseases

Once again, we propose the DDx process is highly affinitive to LTR, especially the listwise approach.

### Case data for clinical decision support system

The case data (= training data) for CDSS is prepared using a literature base [11].

Real World Data (RWD) has not been validated its reliability.

We do not use them as case data for CDSS.

The medical literature includes the following types:

- Medical textbooks
- Medical treatises
- Medical articles
- Case reports

(From now on, referred to as "literature").

Good literature, such as case reports, contains information on confirmed disease(s) and (multiple) differential diseases.

Excellent literature, such as Clinical Problem Solving (CPS), contains information on confirmed disease(s) and (multiple and changing) differential diseases by following the DDx process by experienced physicians [12].

The information discussed in case reports is as follows:

- Symptoms
- Confirmed disease(s)
- Differential diseases (related or to be excluded)

The procedure for making the case data for CDSS is as follows:

1. Select the literature
2. Retrieve the information on cases by text-mining from the literature
3. Convert the retrieved data by text-mining to the symptoms and diseases
4. Store the symptoms and diseases in the database

Technologies have already been developed to automatically text-mining information on the only confirmed disease from the abstracts of case reports [11].

No technology has yet been developed to automatically text-mining information on confirmed disease(s) and (multiple) differential diseases from the body of literature.

No technology has yet been developed to convert retrieved information by text-mining to metadata automatically.

To improve the predictive performance of the CDSS, we propose it is necessary to define strict criteria for symptoms, diseases, and cases.

The criteria we defined for target cases are as follows:

**Rare diseases and difficult-to-diagnose cases that internists and general practitioners may close encounter in actual cases.**

The case data in our system are text-mining data from the literature by us.

### Information retrieval and clinical decision support system

Information Retrieval (IR) is a technique for retrieving information from information resources that match objectives [10].

Google Scholar is a primary IR service that targets scholarly literature on the Internet.

IR systems such as Google Scholar and CDSS have much in common (target data, framework, etc.).

Table 3 shows the Information Retrieval and Clinical Decision Support System.

**Table 3:** Information retrieval and clinical decision support system

Items	Information retrieval (Ex: Google scholar)	Clinical decision support system
Objectives	Get medical literature for target diseases	Get possible diseases
Target data	Medical literature	←
Method of retrieving target data	Web crawlers, etc	Selection by physicians
Framework	Learning-to-rank	←
Input data	Symptoms, Diseases	Symptoms
Output data	Ranking list of useful medical literatures	Ranking list of possible diseases
Evaluation method	Subjective evaluation	Objective evaluation
	Physicians	Case reports
		Evaluation Functions

Retrieval algorithms for IR often use LTR, especially the listwise approach. We propose that CDSS should use several IR technologies (LTR, etc.).

### Conventional clinical decision support systems

Various leading researchers have reported on CDSS based on ML [13–17].

The output of these systems is "predicted diseases." It is "a ranking list of possible diseases." Therefore, these systems are also a type of CDSS based on LTR. However, we assume that the prediction algorithm of these systems uses the pointwise approach. In addition, we assume that the case data of these systems use only confirmed disease information.

We assume that these systems have the following problems:

- The predictive algorithms are LTR with a pointwise approach.
- These algorithms are less affinitive to the DDx process by experienced physicians.
- The case data does not include information on differential diseases.
- These algorithms do not use the relationship between confirmed disease(s) and differential diseases.

### Figures and tables

(See Table 3).

## Design

### Design principles

To address the issues of conventional CDSS, the design principles of our system are as follows:

- The prediction algorithms should be higher affinitive to the DDx process by experienced physicians.
- The case data should include not only information on confirmed disease(s) but also information on differential diseases.
- These algorithms should utilize the relationship between confirmed disease(s) and differential diseases.
- To focus on commonalities between IR and CDSS, utilize various IR technologies for CDSS.

### Library for learning-to-rank

We used TensorFlow and TensorFlow Ranking as our system's Machine Learning (ML) libraries to satisfy the design principles [18, 19].

TensorFlow Ranking is a library for Learning-to-Rank (LTR). The main targets for TensorFlow Ranking are Information Retrieval (IR) systems and Recommendation systems.

For the ranking metrics of LTR, we selected Normalized Discounted Cumulative Gain (NDCG). NDCG is the ranking metric of LTR (listwise approach) [10].

As discussed before, we propose that the calculation algorithm of NDCG is more affinitive to the DDx process by experienced physicians.

For the loss function of LTR, we selected Approximate NDCG loss.

Approximate NDCG loss is an approximation for NDCG. It is a differentiable approximation based on the logistic function [20].

### Case date for learning-to-rank with the listwise approach

The case data for conventional CDSS based on LTR (pointwise approach) has the following information:

- Symptoms
- Confirmed disease

Table 4 shows the Example of case data (pointwise approach).

**Table 4:** Example of case data (pointwise approach)

	Code	Observed symptoms		Code	Diseases
a	Fever	Fever		548	Acute HIV-1 infection
b	Head	Headache			
c	Sore	Sore throat			
d	Myalg	Muscles ache			
e	Fatig	Fatigue	→		
f	Weigh	Weight loss			
g	Arthralg	Arthralgia			
h	Diarrh	Diarrhea			
i	Lymphn	Lymphadenopathy			
j	Mening	Meningitis			
	…				

Based on: case data of our system

These have only information on a confirmed disease.

As discussed before, technologies have already been developed to automatically text-mining this information from the abstracts of case reports.

The case data for our CDSS based on LTR (listwise approach) has the following information:

- Symptoms
- Confirmed disease(s) and these scores
- Differential diseases (related or to be excluded) and these scores

Table 5 shows the Example of case data (listwise approach).

**Table 5:** Example of case data (listwise approach)

	Code	Observed symptoms		Scores	Code	Diseases
a	Fever	Fever		17.078	548	Acute HIV-1 infection
b	Head	Headache		12.086	296	Acute hepatitis
c	Sore	Sore throat		11.250	102	Toxoplasmosis
d	Myalg	Muscles ache		11.000	491	Severe fever with thrombocytopenia syndrome (SFTS)
e	Fatig	Fatigue	→	10.836	391	Osteomyelitis
f	Weigh	Weight loss		10.836	589	Polyneuropathy
g	Arthralg	Arthralgia		10.836	641	Coccidioidomycosis
h	Diarrh	Diarrhea		10.664	627	Cat-scratch disease
i	Lymphn	Lymphadenopathy		10.500	541	Infectious endocarditis
j	Mening	Meningitis		10.414	989	Dengue (hemorrhagic) fever
	…					…

Citation: case data of our system

This information has not only confirmed disease(s) but also differential diseases. In addition, these diseases are assigned a score according to possibility. This information is described not only in the abstracts of literature but also in the bodies.

Thus, the Information Retrieval (IR) system should parse the abstracts and the bodies (See the section Implementation in Additional file 1).

### Figures and tables

(See Tables 4 and 5).

## Evaluation

### Evaluation purposes

The evaluation purposes are to demonstrate the following performance:

- The Machine Learning (ML) performance
  - The ML performance of the system is superior to the conventional system.
- The Differential Diagnostic (DDx) performance
  - The DDx performance of the system is superior to the conventional system.
  - The DDx performance of the system is useful to support the DDx process by physicians.
  - The Clinical Decision Support system (CDSS) is useful in preventing diagnostic errors by physicians.

The notation rules for the loss and evaluation function are as follows:

- Loss function:UPPER CASE (ex: NDCG, MSE, etc.)
- Evaluation function: lower case (ex: ndcg, mse, etc.)

### The compared system

The conventional system we compared was one generation before our system [17].

(From now on, referred to as "the compared system").

In this paper, the other conventional systems we cited were not used for comparison [13–16].

The reasons are:

- The main objective is to propose the prediction algorithm (Learning-to-Rank; listwise approach) for CDSS. In the interest of fairness, the comparison conditions (training data, etc.), except for the algorithm, must be the same. However, these systems' algorithms and training data are not publicly available.
- Each CDSS has different objectives and target diseases.

The compared system also uses Learning-to-Rank (LTR). However, LTR for the compared system is the pointwise approach. The loss function of the compared system is Mean Squared Error (MSE).

### Evaluation criteria for differential diagnostic performance

As evaluation criteria for DDx performance, we focused on confirmed diseases (or related diseases) that should be ranked in the top 10th predicted diseases.

The reasons are:

- The DDx process by physicians is a kind of incomplete information game [21]. The acquired information, thoughts, and knowledge may contain mistakes or omissions in this process [22]. In today's CDSS, the main objective is a Decision Support System, not a Diagnosis System.
- Physicians decide the final confirmed disease(s) by themselves, using the predicted diseases of CDSS as a reference.

### Case selection criteria for evaluation of differential diagnostic performance

In previous articles, cases for evaluation of DDX performance are often actual cases [23].

However, they should be validated cases with case reports, etc.

The reasons are:

- Our main target diseases are rare diseases and difficult-to-diagnose cases that internists and general practitioners may close encounter in clinical practice**.** However, the probability of encountering these diseases is low.
- For correct evaluation, it is important to evaluate with validated cases.

"The New England Journal of Medicine (NEJM)" publishes many excellent case reports that fit these purposes.

Therefore, we used case reports from NEJM to evaluate the DDx performance of the CDSS.

## Evaluation: machine learning performance

### Evaluation method

The Machine Learning (ML) performance of Clinical Decision Support System (CDSS) valuated was as follows:

- Learning curves
- Value of evaluation function

The data used to evaluate the ML performance were the case data we collected. The number of case data was around 26,000.

We evaluated the ML performance on k-fold cross-validation (k = 5).

In the interest of fairness, the comparison conditions (training data, validated data, hyperparameters. etc.), except for the loss function, were the same.

### Evaluation results and discussion

**Fig. 2:**
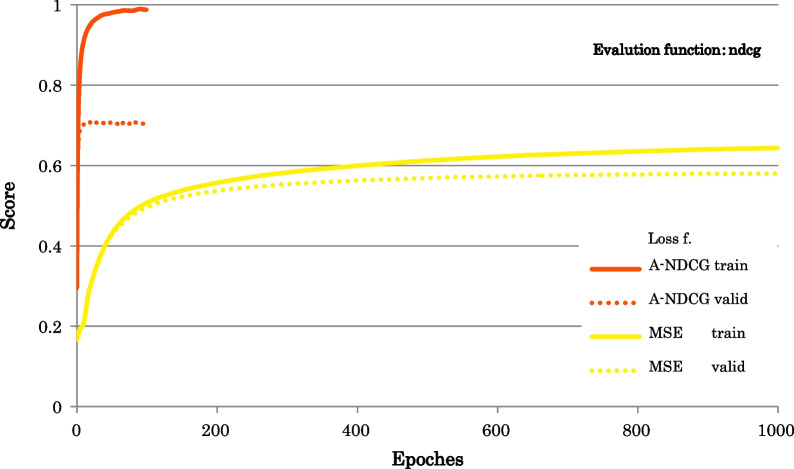
Learning curves of ndcg. Evaluation function: ndcg; Loss functions: A-NDCG: Approximate NDCG loss, MSE: mean squared error

Figure 2 shows the Learning curves of ndcg.

**Fig. 3:**
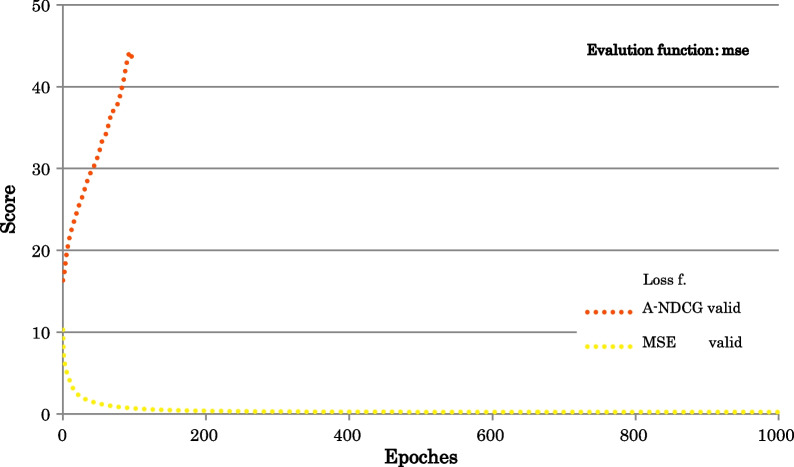
Learning curves of mse. Evaluation function: mse; Loss functions: A-NDCG: Approximate NDCG loss, MSE: mean squared error

Figure 3 shows the Learning curves of mse.

Table 6 shows the Value of evaluation functions.

**Table 6:** Value of evaluation functions

Loss functions	Evaluation functions			
	ndcg	ndcg@5	ndcg@10	ndcg@20
A-NDCG	0.7098	0.6205	0.6485	0.6680
MSE	0.5835	0.4470	0.4845	0.5139

The findings from the results of the Learning curves of ndcg are as follows:

- The number of epochs in training was larger for MSE.
- However, the training time was longer for A-NDCG.
- The memory space requirement was larger for A-NDCG.
- We found that the prediction model with A-NDCG tended to overfit.

The findings from the results of the Learning curves of mse are as follows:

- For LTR, we found that mse was not a suitable evaluation function.

The findings from the value of evaluation functions are as follows:

- The value of the evaluation functions was consistently higher for A-NDCG.

The ML performance differences between A-NDCG and MSE were very significant.

We tested ML performance tuning with the following techniques:

- Hyperparameters tuning with Bayesian optimization
- Change of the neural network configuration
  - Number of layers
  - Activation function
  - Optimizer algorithm

However, the effect of improved ML performance was small.

As the loss function, we tested the Gumbel approximate NDCG loss, a member of the Approximate NDCG loss family [24].

However, due to the memory space requirement for training, the effect of improving ML performance was insignificant.

### Figures and tables

(See Figs. 2, 3 and Table 6).

## Evaluation: differential diagnosis performance

### Evaluation method

The Differential Diagnosis (DDx) performance of Clinical Decision Support System (CDSS) evaluated was as follows:

- Predicted diseases

The following data are available in the Additional file 2:

- Inputted symptoms and predicted diseases
- Inputted symptoms and the target disease's ranking

The cases we selected for evaluation from "The New England Journal of Medicine (NEJM)" were as follows:

- Disease with characteristic symptoms
  - Acute intermittent porphyria [25]
- Difficult-to-diagnose case with few characteristic symptoms
  - Acute HIV-1 infection [26]
- Case with diagnostic errors
  - Subacute bacterial endocarditis caused by bartonella [27]

We have selected the cases we consider typically, following our case selection criteria.

The steps of the evaluation process with case reports were as follows:

1. Pick up diseases (confirmed and differential) from the case report.
2. Pick up symptoms, etc., from the case report.
3. Translate symptoms of the case report into symptoms of the CDSS.
4. Input symptoms into the CDSS.
5. Compare predicted diseases of the CDSS with diseases of the case report.

The training data of both CDSS to evaluate the DDx performance were the case data we collected. The number of case data was around 26,000.

In the interest of fairness, the comparison conditions (training data, hyperparameters. etc.), except for the loss function, were the same.

In addition, these cases were not used as training data.

### Evaluation results and discussion

#### Disease with characteristic symptoms

We evaluated the Differential Diagnostic (DDx) performance of the disease with characteristic symptoms.

The DDx of these diseases is manageable to a conventional Clinical Decision Support System (CDSS).

The case we used was acute intermittent porphyria (AIP) [25].

In both systems, the confirmed disease, in this case, is as follows:

- Acute intermittent porphyria (AIP)

Table 7 shows the Predicted diseases: case of the acute intermittent porphyria.

**Table 7:** Predicted diseases: case of the acute intermittent porphyria

	A-NDCG	MSE
1	Acute intermittent porphyria	Acute intermittent porphyria
2	Diabetic coma imminent state	Enterohemorrhagic e. coli (EHEC) infection
3	Pesticide poisoning, Organophosphate toxicity	Visceral rupture
4	Lead poisoning (almost chronic)	Fibromyalgia (fibrositis)
5	Heat stroke (hyperthermia)	Cancerous peritonitis
6	Cytomegalovirus infection	Withdrawal symptoms of alcohol and drugs
7	Visceral rupture	Colorectal cancer
8	Hyponatremia	Irritable bowel syndrome, Functional dyspepsia (FD)
9	Portal vein obstruction	Drugs (laxatives, etc.)
10	Acetaminophen poisoning	Eating disorder
	…	

Cited case: Acute intermittent porphyria [25]

Loss functions: *A-NDCG*: Approximate NDCG loss, *MSE*: Mean Squared Error

In both systems, the predicted ranking of confirmed disease was 1st.

In the predicted diseases of our system, the excluded diseases for AIP (ex: lead poisoning) were listed at the top of the list [28, 29].

In this case, the predicted diseases of our system provided useful information for the DDx process by physicians.

Regarding "Inputted symptoms and the target disease's ranking," in both systems, at the point where the characteristic symptoms (hyponatremia and abnormal liver function) were inputted, the final confirmed disease was listed at the top of the list.

For the DDx of diseases with characteristic symptoms, we suppose that the DDx performances of both systems are not significantly different.

#### Difficult-to-diagnose case with few characteristic symptoms

We evaluated the Differential Diagnosis (DDx) performance of the difficult-to-diagnose case with few characteristic symptoms.

The DDx of these diseases is difficult to conventional Clinical Decision Support System (CDSS).

The case we used was acute HIV-1 infection [26].

In HIV infection, acute meningitis symptoms may develop at the time of initial infection [30].

In both systems, the related diseases, including the confirmed disease, in this case, are as follows:

- Acute HIV-1 infection
- Acute viral meningitis

Therefore, these diseases were also defined as related diseases to confirmed diseases.

Table 8 shows the Predicted diseases: case of the acute HIV-1 infection.

**Table 8:** Predicted diseases: case of the acute HIV-1 infection

	A-NDCG	MSE
1	Acute HIV-1 infection	Epidemic hepatitis A
2	Polyneuropathy	Acute Q fever
3	Acute viral meningitis	Acute pharyngitis
4	West Nile fever	Polyneuropathy
5	Cat-scratch disease	Lymphocytic choriomeningitis
6	Acute Q fever	Herpes labialis
7	Epidemic hepatitis A	Side effects of interferon
8	Chronic fatigue syndrome	Sepsis
9	Sepsis	Chronic fatigue syndrome
10	Toxoplasmosis	Retropharyngeal infection
	…	

Cited case: Acute HIV-1 infection [26]

Loss functions: *A-NDCG*: Approximate NDCG loss; *MSE*: Mean Squared Error

In our system, the predicted rankings of related diseases were as follows:

1st: Acute HIV-1 infection.

3rd: Acute viral meningitis.

However, in the compared system, the predicted rankings of related diseases were less than the 20th.

Regarding "Inputted symptoms and the target disease's ranking," in our system, at the point where few symptoms were inputted, related diseases were listed at the top of the list.

In this case, many of these symptoms are common in other diseases.

For the DDx of difficult-to-diagnose cases with few characteristic symptoms, we suppose that DDx performance of our system is superior.

#### Case with diagnostic errors

Cognitive biases, such as confirmation bias, are among the most frequent causes of diagnostic errors [31].

Clinical Decision Support System (CDSS) is useful for preventing diagnostic errors.

We evaluated the Differential Diagnostic (DDx) performance of a case with diagnostic errors. The system used for the evaluation of this case was only our system.

The final confirmed disease of the case was subacute bacterial endocarditis caused by bartonella [27].

The title of the case report is "Copycat." In this case, this patient had a history of HCV infection. Initially, due to confirmation bias, the case report's authors did not focus on the characteristic symptoms of endocarditis (heart murmur, purpura, etc.) but this HCV infection. As a result, they reported the misdiagnosed case as mixed cryoglobulinemia by HCV.

In our system, the related diseases, including the confirmed disease, in this case, are as follows:

- Subacute bacterial endocarditis (SBE)
- Acute bacterial endocarditis
- Infectious endocarditis

Therefore, these diseases were also defined as related diseases to confirmed diseases.

In addition, the misdiagnosed disease is as follows:

- Mixed cryoglobulinemia

Table 9 shows the Predicted diseases: case of the subacute bacterial endocarditis caused by bartonella: In progress.

**Table 9:** Predicted diseases: case of the subacute bacterial endocarditis caused by bartonella: In progress

	Predicted diseases	Classification
1	Zieve syndrome	
2	Disseminated intravascular coagulation	
3	Chronic hepatitis	
4	Wilson's disease	
5	Acute hepatitis	
6	Hepatic amyloidosis	
7	Infectious endocarditis	Related disease
8	(Compensated/uncompensated) liver cirrhosis	
9	Subacute bacterial endocarditis	Related disease
10	Gastric cancer	
	…	

Case: Subacute bacterial endocarditis caused by bartonella [27]

Loss functions: *A-NDCG*: Approximate NDCG loss; In progress: Number of inputted symptoms = 9

Table 10 shows the Predicted diseases: case of the subacute bacterial endocarditis caused by bartonella: Final.

**Table 10:** Predicted diseases: case of the subacute bacterial endocarditis caused by bartonella: Final

	Predicted diseases	Classification
1	*Mixed cryoglobulinemia*	Misdiagnosed disease
2	Chronic hepatitis	
3	Subacute bacterial endocarditis	Related disease
4	Hepatic amyloidosis	
5	Rapidly progressive glomerulonephritis syndrome	
6	Acute bacterial endocarditis	Related disease
7	Infectious endocarditis	Related disease
8	Polyarteritis nodosa	
9	Autoimmune hemolytic anemia	
10	Disseminated intravascular coagulation	
	…	

Cited case: Subacute bacterial endocarditis caused by bartonella [27]

Loss functions: *A-NDCG*: Approximate NDCG loss; Final: Number of inputted symptoms = 18

In the final predicted diseases (Table 10), the misdiagnosed disease was ranked 1st. The cause was the information by confirmation bias. Nevertheless, the related diseases were ranked in the top 10.

In the progress predicted diseases (Table 9), the related diseases were ranked in the top 10.

Despite the biased information, the system listed the related disease at the top. In the DDx process by physicians, if they had this information, we assume that their differential disease list would include not only HIV infection but also SBE.

We propose that the CDSS, including our system, will prevent diagnostic errors by physicians.

### Figures and tables

(See Tables 7, 8, 9, 10).

## Conclusion

This paper discusses the design, implementation, and evaluation of our Clinical Decision Support System (CDSS) based on Learning-to-Rank (LTR) with the listwise approach.

### Evaluation results

We evaluated Machine Learning (ML) performance and Differential Diagnosis (DDx) performance.

The ML and DDx performance of our system (listwise approach: A-NDCG) was higher than that of the compared system (pointwise approach: MSE).

In terms of both ML and DDx performance, we have demonstrated that the CDSS is useful for physicians to support DDx and prevent diagnostic errors.

### Differential diagnosis process by physicians and learning to rank by machines

The prediction algorithm of our system is Learning-to-Rank (LTR) with the listwise approach. The Differential Diagnosis (DDx) process by physicians is an iterative process with Recalling, Refining, and Ranking differential diseases.

### Case data and information retrieval

Our system's case data (= training data) and predicted results are almost the same data structure.

Table 11 shows the Case data and predicted results of our system.

**Table 11:** Case data and predicted results of our system

	Case data (= training data)	Predicted results
X: explanatory variables	Observed symptoms	Inputted symptoms
y: explained variables	Confirmed disease(s) and those score(s) & Differential diseases (related or to be excluded) and those scores	Predicted diseases and those scores

When experienced physicians validate the predicted diseases, for feedback on validation results to the predictive model, we propose that the results of our system (listwise approach: A-NDCG) are more pertinent than the results of the compared system (pointwise approach: MSE).

As discussed before, no technology has yet been developed to automatically optimize case data for a listwise approach.

Therefore, we had to do these tasks manually (and by only one physician).

As a result, due to his knowledge and thought, our system may have both bias and outstanding performance.

For the practical application of Clinical Decision Support System (CDSS), we propose that developing the following Information Technologies (IT) is necessary:

- Technology for predicting diseases, such as Learning-to-Rank (LTR)
- Technology for text-mining information on diseases from literatures
- Technology for converting text-mining data to the symptoms and diseases

For this purpose, using Information Retrieval (IR) technologies is effective.

### Potentials for clinical decision support system

According to our experience and knowledge, we presume that Clinical Decision Support System (CDSS), including our system, has the following potential:

- Recall rare diseases
- Support differential diagnoses for difficult-to-diagnose cases
- Prevent diagnostic errors

### Evolution into explainable clinical decision support system

We suppose our system can evolve into an Explainable Clinical Decision Support System (X-CDSS) [32].

The reasons for this are as follows:

- The affinity between Differential Diagnosis (DDx) processes by experienced physicians and LTR with the listwise approach
- The similarity between case data (= training data) and predicted results
- The simple neural network
  - The number of internal hiding layers is one.
  - The number of learnable times (epochs) is relatively small.

We will continue to develop the Ultimate Clinical Decision Support System (U-CDSS).

### Figures and tables

(See Table 11).

## Supplementary Information

**Additional file 1**: All of implementation

**Additional file 2**: A part of evaluation results of differential diagnosis performance

## Data Availability

The datasets used and/or analyzed during the current study are available from the corresponding author on reasonable request. Contact information is as follow: - https://www.diagnosis.or.jp/. - mailto: ai.diagnosis.2021@gmail.com.

## Abbreviations

CDSS: Clinical decision support system
DDSS: Diagnosis decision support system
RD: Rare diseases
IR: Information retrieval
LTR: Learning to rank
ML: Machine learning
DDx: Differential diagnosis
NDCG: Normalized discounted cumulative gain
A-NDCG: Approximate NDCG, as a loss function
MSE: Mean squared error, as a loss function
ndcg: NDCG, as an evaluation function
mse: Mean squared error, as an evaluation function
