# Supplementary material for: Design, implementation, and evaluation of the computer-aided clinical decision support system based on learning-to-rank: collaboration between physicians and machine learning in the differential diagnosis process
Source: BMC Med Inform Decis Mak. 2023 Feb 2;23:26. doi: 10.1186/s12911-023-02123-5 (PMC9896739; doi:10.1186/s12911-023-02123-5)
Supplement: Supplementary file 1 — Additional file 1: All of implementation [file 12911_2023_2123_MOESM1_ESM.pdf]

Title

Implementation

Title of main manuscript

Design, Implementation, and Evaluation of the Computer-aided Clinical Decision Support System based on Learning-to-Rank: Collaboration between physicians and machine learning in the differential diagnosis process

Version

- 2023013001

Contact Information

- *https:/www.diagnosis.or.jp/*
- *mailto: ai.doagnosis.2021@gmail.com*

Implementation

Database

Overview

The contents of the database are as follows:

- Symptoms master data
- Diseases master data
- Case data

Symptom master data

The symptom master data is used as "inputted symptoms."

Information in the symptom master data is as follows:

- Subjective symptoms
- Objective findings
- Physical findings
- Laboratory test results
- Imaging tests results
- Other Information

We used a qualitative expression, not a numerical one, for quantitative data.
We used an imaging finding, not imaging data, for imaging test results.
We did not use quantity or quality, except for special findings (ex: masses, atelectasis, pleural effusion, pneumothorax, etc.).

Table 1 shows the Example of expression in the symptom master data.

One code defines multiple similar symptoms. The reason is to support the input of symptoms by physicians.
The code system and the name of the symptoms in the symptom master data are based on our specifications.
The number of codes in the symptom master data is 583.
The total number of symptoms is approximately 1,200.

Table 2 shows the Example of the symptom master data.

Disease master data

The disease master data is used as "predicted diseases."

One record includes confirmed disease(s) and differential diseases (related or to be excluded). The reason is to support the differential diagnosis by physicians.
The code system and name of diseases in the disease master data are based on our specifications.
The number of records in the disease master data is 1,000.
The total number of diseases is approximately 6,700.

Table 3 shows the Example of the disease master data.

Case data

The case data is used as "training data."

As discussed in the main manuscript, no technology has yet been developed to do these tasks automatically. Therefore, we had to do these tasks manually.
We always weighted the scores for confirmed diseases by α (e.g., α = 10).
The number of data for the case data is around 26,000.
We converted our case data to a data-frame format.

Table 4 shows the Example of the case date (data-frame format).

Development and execution environment

Table 5 shows the Development and execution environment of the system.

Neural network configuration

The notation rules for the loss and evaluation function are as follows:

- Loss function: UPPER CASE (ex: NDCG, MSE, etc.)
- Evaluation function: lower case (ex: ndcg, mse, etc.)

Table 6 shows the Neural network configuration of the system.

Implementation code

Table 7 shows the Code fragment of the system.

Figures and Tables

Table 1 Example of expression in the symptom master data

Type of expression	Type of symptom	Example of item	Example of expression
Qualitative expression	Physical findings	Fever	high / low
		Respiratory rate	more / less
	Laboratory test results	Blood glucose level	high / low
		White blood cell count	more / less
Image findings	Imaging test results	Chest radiograph	Abnormal chest radiograph
		Bone radiograph	Abnormal bone radiograph

Table 2 Example of the symptom master data

Code	Value
fever	Fever; high body temperature; hyperthermia
head	Headache; nuchal pain; temporal headache; morning headache
sore	Sore throat; pharyngeal erythema; pharyngeal erosion; throat pain
myalg	Myalgia; muscle (s) ache; muscle pain
fatig	Fatigue; easy fatigued/fatigability; tiredness; lassitude; feel tired
weigh	Weight loss; the clothes appear to fit loosely
arthralg	Arthralgia (take no account of the property or the location)
diarrh	Diarrhea; loose stool; pass a loose bowel movement
lymphn	Lymphadenopathy/adenopathy (take no account of the property/location)
…	

Citation: symptom master data of our system

Table 3 Example of the disease master data

Code	Name
R42	Acute porphyria (acute intermittent porphyria, variegate porphyria, Hereditary coproporphyria), …
R790	Diabetic coma imminent state, hyperosmolar hyperglycemic syndrome (HHS), …
R535	Pesticide poisoning, organophosphate toxicity …
R876	Lead poisoning (almost chronic), aromatic hydrocarbons intoxication, halocarbon poisoning, …
R117	Cytomegalovirus infection, CMV syndrome (note: AIDS and other opportunistic infections), post-cardiac surgery (extracorporeal circulation) syndrome, …
R920	Visceral rupture, bleeding or hematoma (cause of anemia, faint, or falling down) infarction, …
R765	Hyponatremia, …
R499	Portal vein obstruction, portal vein thrombosis, pylephlebitis, portal vein gas (carefully evaluate the existence of intestinal necrosis), …
R957	Acetaminophen poisoning, …
…	

Citation: disease master data of our system

Table 4 Example of the case date (data-frame format)

case	X					Y				
	0	1		581	582	0	1		998	999
0	1	0		0	0	0.000	1.442		0.385	5.290
1	0	0		0	0	0.000	0.000		10.000	6.250
			…					…		
26384	0	0		0	0	1.282	0.961		3.460	1.923

Citation: case data of our system

Table 5 Development and execution environment of the system

Type	
**Programming Language**	Python 3.8.x
**Software Libraries**	TensorFlow 2.7.x<br>TensorFlow Ranking 0.5.x
**Web Application Framework**	Flask 2.0.x
**Cloud Computing Services**	Google Cloud Platform<br>App Engine<br>AI Platform Engine<br>Firebase Authentication

Table 6 Neural network configuration of the system

Items	Values		Notes
**Input layer**	583	units	Number of symptom codes
**Hidden layer**	1	layer	
	1024	units	
**Output layer**	1000	units	Number of disease records
**Loss functions**	Approximate NDCG loss (A-NDCG)		Our system
	Mean Squared Error (MSE)		Compared system
**Evaluation functions**	ndcg		
	ndcg@k		
	mse		

Table 7 Code fragment of the system

```
import tensorflow as tf
import tensorflow_ranking as tfr

N_SYMPTOM_CODE = 583
N_DISEASE_CODE = 1000

HIDDEN_UNITS = 1024
ACTIVATION = tf.keras.activations.relu
OPTIMIZER = tf.keras.optimizers.Adam()

# LOSS = tf.keras.losses.MeanSquaredError()
LOSS = tfr.keras.losses.ApproxNDCGLoss()

METRICS = [
    tf.keras.metrics.MeanSquaredError(name="mse"),
    tfr.keras.metrics.NDCGMetric(name="ndcg"),
    tfr.keras.metrics.NDCGMetric(name="ndcg_1", topn=1),
    tfr.keras.metrics.NDCGMetric(name="ndcg_3", topn=3),
    tfr.keras.metrics.NDCGMetric(name="ndcg_5", topn=5),
    tfr.keras.metrics.NDCGMetric(name="ndcg_10", topn=10),
    tfr.keras.metrics.NDCGMetric(name="ndcg_20", topn=20),
]

# BATCH_SIZE = 4096    # MSE
BATCH_SIZE = 512    # ApproxNDCG

# EPOCHS = 900    # MSE
EPOCHS = 80    # ApproxNDCG
```

```
def train(X_train, y_train, X_test, y_test):

    model = tf.keras.models.Sequential()

    model.add(tf.keras.layers.InputLayer(input_shape=(N_SYMPTOM_CODE,)))
    model.add(tf.keras.layers.Dense(HIDDEN_UNITS))
    model.add(tf.keras.layers.Activation(ACTIVATION))
    model.add(tf.keras.layers.Dense(N_DISEASE_CODE))

    model.compile(OPTIMIZER, LOSS, METRICS)

    model.fit(X_train, y_train,
              batch_size=BATCH_SIZE,
              epochs=EPOCHS,
              validation_data=(X_test, y_test))

    return model
```
