# Supplementary material for: Design, implementation, and evaluation of the computer-aided clinical decision support system based on learning-to-rank: collaboration between physicians and machine learning in the differential diagnosis process
Source: BMC Med Inform Decis Mak. 2023 Feb 2;23:26. doi: 10.1186/s12911-023-02123-5 (PMC9896739; doi:10.1186/s12911-023-02123-5)
Supplement: Supplementary file 2 — Additional file 2: A part of evaluation results of differential diagnosis performance [file 12911_2023_2123_MOESM2_ESM.pdf]

Title

Additional information of evaluation results

Title of main manuscript

Design, Implementation, and Evaluation of the Computer-aided Clinical Decision Support System based on Learning-to-Rank: Collaboration between physicians and machine learning in the differential diagnosis process

Version

- 2023013001

Contact Information

- *https:/www.diagnosis.or.jp/*
- *mailto: ai.doagnosis.2021@gmail.com*

Evaluation: Differential Diagnosis performance

Evaluation results and Discussion

Disease with characteristic symptoms

Cited case:

- Acute intermittent porphyria [25]

Table 1 shows the Inputted symptoms and predicted diseases: case of the acute intermittent porphyria.

Table 2 shows the Inputted symptoms and the target disease's ranking: case of the acute intermittent porphyria.

Difficult-to-diagnose case with few characteristic symptoms

Cited case:

- Acute HIV-1 infection [26]

Table 3 shows the Inputted symptoms and predicted diseases: case of the acute HIV-1 infection.

Table 4 shows the Inputted symptoms and the target disease's ranking: case of the acute HIV-1 infection.

Case with diagnostic errors

Cited case:

- Subacute bacterial endocarditis caused by bartonella [27]

Table 5 shows the Inputted symptoms and predicted diseases: case of the subacute bacterial endocarditis caused by bartonella: In progress: n = 5.

Table 6 shows the Inputted symptoms and predicted diseases: case of the subacute bacterial endocarditis caused by bartonella: In progress: n = 9.

Table 7 shows the Inputted symptoms and predicted diseases: case of the subacute bacterial endocarditis caused by bartonella: In progress: n = 14.

Table 8 shows the Inputted symptoms and predicted diseases: case of the subacute bacterial endocarditis caused by bartonella: Final: n = 18.

Figures and Tables

Table 1 Inputted symptoms and predicted diseases: case of the acute intermittent porphyria

	Inputted symptoms		Predicted diseases				
				A-NDCG			MSE
a	Abdominal pain		1	1.41	**Acute intermittent porphyria**	9.25	**Acute intermittent porphyria**
b	Constipation		2	1.21	Diabetic coma imminent state	8.17	Enterohemorrhagic e. coli (EHEC) infection
c	Hyponatremia		3	0.56	Pesticide poisoning, Organophosphate toxicity	7.64	Visceral rupture
d	Abdominal tenderness		4	0.51	Lead poisoning (almost chronic)	7.56	Fibromyalgia (fibrositis)
e	Elevated serum liver enzyme		5	0.45	Heat stroke (hyperthermia)	7.53	Cancerous peritonitis
f	Tachycardia	→	6	0.31	Cytomegalovirus infection	7.29	Withdrawal symptoms of alcohol and drugs
g	Photophobia		7	0.25	Visceral rupture	7.28	Colorectal cancer
h	Thirsty		8	0.16	Hyponatremia	7.26	Irritable bowel syndrome, Functional dyspepsia (FD)
i	Borborygmus		9	0.13	Portal vein obstruction	7.23	Drugs (laxatives, etc.)
j	Dehydration		10	0.12	Acetaminophen poisoning	7.07	Eating disorder
k	Confusion				…		

Cited case: Acute intermittent porphyria [25]

Loss functions: A-NDCG: Approximate NDCG loss

MSE: Mean Squared Error

Table 2 Inputted symptoms and the target disease's ranking: case of the acute intermittent porphyria

	Inputted symptoms		Ranking	
		Target disease	AIP	
		Loss functions	A-NDCG	MSE
a	Abdominal pain		---	---
b	Constipation	→	---	---
c	Hyponatremia		1	4
d	Abdominal tenderness	→→	1	19
e	Elevated serum liver enzyme		1	4
f	Tachycardia	→→→	1	1
g	Photophobia		1	1
h	Thirsty	→→→→	1	1
i	Borborygmus		1	1
j	Dehydration	→→→→→	1	1
k	Confusion		1	1

Cited case: Acute intermittent porphyria [25]
Target disease: AIP: acute intermittent porphyria
Loss functions: A-NDCG: Approximate NDCG loss
MSE: Mean Squared Error
---: 20th and lower

Table 3 Inputted symptoms and predicted diseases: case of the acute HIV-1 infection

	Inputted symptoms			Predicted diseases		
				A-NDCG		MSE
a	Fever		1	1.61	**Acute HIV-1 infection**	10.16	Epidemic hepatitis A
b	Headache		2	1.51	Polyneuropathy	9.93	Acute Q fever
c	Sore throat		3	0.91	**Acute viral meningitis**	9.75	Acute pharyngitis
d	Consciousness indistinctness		4	0.88	West Nile fever	9.66	Polyneuropathy
e	Chills		5	0.77	Cat-scratch disease	9.28	Lymphocytic choriomeningitis
f	Muscles ache		6	0.46	Acute Q fever	9.17	Herpes labialis
g	Swallowing pain	→	7	0.23	Epidemic hepatitis A	8.99	Side effects of interferon
h	Pharyngolaryngeal abnormality		8	0.21	Chronic fatigue syndrome	8.86	Sepsis
i	Aphasia		9	0.13	Sepsis	8.74	Chronic fatigue syndrome
j	Apraxia		10	0.12	Toxoplasmosis	8.73	Retropharyngeal infection
k	Fatigue				...		
l	Muscle weakness						
m	Anorexia						
n	Weight loss						
o	Dementia						

Cited case: Acute HIV-1 infection [26]

Loss functions: A-NDCG: Approximate NDCG loss

MSE: Mean Squared Error

Table 4 Inputted symptoms and the target disease's ranking: case of the acute HIV-1 infection

	**Inputted symptoms**		**Ranking**			
		**Target diseases**	HIV-1		AVM	
		**Loss functions**	A-NDCG	MSE	A-NDCG	MSE
a	Fever		---	---	---	---
b	Headache		---	---	9	1
c	Sore throat		14	---	10	---
d	Consciousness indistinctness	→	---	---	3	10
e	Chills		---	---	2	9
f	Muscles ache	→→	---	---	3	---
g	Swallowing pain		14	---	5	---
h	Pharyngolaryngeal abnormality	→→→	---	---	9	---
i	Aphasia		---	---	6	---
j	Apraxia	→→→→	---	---	2	19
k	Fatigue		---	---	2	---
l	Muscle weakness	→→→→→	12	---	2	---
m	Anorexia		12	---	1	---
n	Weight loss		1	---	3	---
o	Dementia		1	---	3	---

Cited case: Acute HIV-1 infection [26]

Target diseases: HIV-1: acute HIV-1 infection

AVM: acute viral meningitis

Loss functions: A-NDCG: Approximate NDCG loss

MSE: Mean Squared Error

---: 20th and lower

Table 5 Inputted symptoms and predicted diseases: case of the subacute bacterial endocarditis caused by bartonella: In progress: n = 5

	Inputted symptoms				Predicted diseases	Classification
a	Weight loss		1	0.78	Hodgkin lymphoma	
b	Itching		2	0.60	Heart tumor	
c	Fatigue		3	0.41	Mitral valve stenosis	
d	Tachypnea		4	0.32	**Infectious endocarditis**	Related disease
e	Heart murmur		5	0.17	Aortic stenosis	
			6	0.16	Acute Q fever	
			7	0.04	Hyperthyroidism	
			8	0.03	Immunoblastic lymphadenopathy	
		→	9	0.03	Hepatic amyloidosis	
			10	0.03	Hashimoto's disease and autoimmune thyroiditis	
					…	

Cited case: Subacute bacterial endocarditis caused by bartonella [27]

Loss functions: A-NDCG: Approximate NDCG loss

In progress: Number of inputted symptoms = 5

Table 6 Inputted symptoms and predicted diseases: case of the subacute bacterial endocarditis caused by bartonella: In progress: n = 9

	Inputted symptoms				Predicted diseases	Classification
a	Weight loss		1	1.61	Zieve syndrome	
b	Itching		2	1.35	Disseminated intravascular coagulation	
c	Fatigue		3	0.94	Chronic hepatitis	
d	Tachypnea		4	0.77	Wilson's disease	
e	Heart murmur		5	0.76	Acute hepatitis	
f	Purpura		6	0.69	Hepatic amyloidosis	
g	Anemia		7	0.59	**Infectious endocarditis**	Related disease
h	Elevated serum liver enzyme		8	0.59	(Compensated / uncompensated) liver cirrhosis	
i	Hyperbilirubinemia	→	9	0.53	**Subacute bacterial endocarditis**	Related disease
			10	0.53	Gastric cancer	
					…	

Cited case: Subacute bacterial endocarditis caused by bartonella [27]

Loss functions: A-NDCG: Approximate NDCG loss

In progress: Number of inputted symptoms = 9

Table 7 Inputted symptoms and predicted diseases: case of the subacute bacterial endocarditis caused by bartonella: In progress: n = 14

	Inputted symptoms				Predicted diseases	Classification
a	Weight loss		1	1.78	**Subacute bacterial endocarditis**	Related disease
b	Itching		2	1.35	Hepatic amyloidosis	
c	Fatigue		3	1.32	Chronic hepatitis	
d	Tachypnea		4	1.29	**Infectious endocarditis**	Related disease
e	Heart murmur		5	1.16	Disseminated intravascular coagulation	
f	Purpura		6	1.15	*Mixed cryoglobulinemia*	Misdiagnosed disease
g	Anemia		7	1.10	**Acute bacterial endocarditis**	Related disease
h	Elevated serum liver enzyme		8	0.99	Idiopathic (or secondary) retroperitoneal fibrosis	
i	Hyperbilirubinemia	→	9	0.98	Tuberculosis or syphilis	
j	ESR elevation		10	0.90	Thrombotic thrombocytopenic purpura	
k	RA/RF positive				…	
l	Hematuria					
m	Bacteriuria					
n	Proteinuria					

Cited case: Subacute bacterial endocarditis caused by bartonella [27]

Loss functions: A-NDCG: Approximate NDCG loss

In progress: Number of inputted symptoms = 14

Table 8 Inputted symptoms and predicted diseases: case of the subacute bacterial endocarditis caused by bartonella: Final: n = 18

	Inputted symptoms				Predicted diseases	Classification
a	Weight loss		1	2.70	*Mixed cryoglobulinemia*	Misdiagnosed disease
b	Itching		2	2.62	Chronic hepatitis	
c	Fatigue		3	2.03	**Subacute bacterial endocarditis**	Related disease
d	Tachypnea		4	1.48	Hepatic amyloidosis	
e	Heart murmur		5	1.47	Rapidly progressive glomerulonephritis syndrome	
f	Purpura		6	1.34	**Acute bacterial endocarditis**	Related disease
g	Anemia		7	1.27	**Infectious endocarditis**	Related disease
h	Elevated serum liver enzyme		8	0.94	Polyarteritis nodosa	
i	Hyperbilirubinemia	→	9	0.78	Autoimmune hemolytic anemia	
j	ESR elevation		10	0.75	Disseminated intravascular coagulation	
k	RA/RF positive				…	
l	Hematuria					
m	Bacteriuria					
n	Proteinuria					
o	Hypocomplementemia					
p	Angiitis					
q	Nephritis					
r	Animal Origin Infections					

Cited case: Subacute bacterial endocarditis caused by bartonella [27]

Loss functions: A-NDCG: Approximate NDCG loss

Final: Number of inputted symptoms = 18
